# Baicalin Protects Vascular Tight Junctions in Piglets During *Glaesserella parasuis* Infection

**DOI:** 10.3389/fvets.2021.671936

**Published:** 2021-06-25

**Authors:** Yu Liu, Xiaoyi Li, Zhaoran Zhang, Jiacheng Zhang, Jianfeng Xu, Yinsheng Qiu, Chun Ye, Shulin Fu, Zhongyuan Wu, Chien-An Andy Hu

**Affiliations:** ^1^Hubei key Laboratory of Animal Nutrition and Feed Science, School of Animal Science and Nutritional Engineering, Wuhan Polytechnic University, Wuhan, China; ^2^Biochemistry and Molecular Biology, University of New Mexico School of Medicine, Albuquerque, NM, United States

**Keywords:** vascular barrier, tight junctions, baicalin, *Glaesserella parasuis*, piglets

## Abstract

*Glaesserella parasuis* (*G. parasuis*) can cause Glässer's disease and severely affect swine industry worldwide. This study is an attempt to address the issue of the capability of *G. parasuis* to damage the vascular barrier and the effects of baicalin on vascular tight junctions (TJ) in order to investigate the interactions between the pathogen and the porcine vascular endothelium. Piglets were challenged with *G. parasuis* and treated with or without baicalin. The expressions of vascular TJ genes were examined using RT-PCR. The distribution patterns of TJ proteins were detected by immunofluorescence. The involved signaling pathways were determined by Western blot assays on related proteins. *G. parasuis* can downregulate TJ expression and disrupt the distribution of TJ proteins. Baicalin can alleviate the downregulation of vascular TJ mRNA, maintain the distribution, and prevent the abnormalities of TJ. These results provide ample evidence that baicalin has the capacity to protect vascular TJ damaged by *G. parasuis* through inhibiting PKC and MLCK/MLC pathway activation. As a result, baicalin is a promising candidate for application as a natural agent for the prevention and control of *G. parasuis* infection.

## Introduction

The vascular endothelial barrier is located at the interface between interstitial tissues and blood. The basic function of the barrier is to regulate the flow of fluid and solutes between the surrounding tissue and the blood (1). Adhesive junctions, including tight junctions (TJ) and adherens junctions between endothelial cells, form the paracellular pathway of vascular barrier (2). TJ are complex structure of different proteins, which are fundamental for the integrity of the vascular endothelial barrier (2). TJ skeleton is formed of integral membrane proteins, such as occludin, claudins, and junctional adhesion molecules (JAMs). Peripheral membrane proteins zonula occludens (ZOs) are the bridge between cytoskeleton and transmembrane proteins (3). The stability of its function requires the coordination of the multiple proteins (4). TJ contribute to prevent pathogens from entering the organisms from the external environment, which makes TJ the initial target of pathogens. Pathogens and inflammatory conditions degrade TJ integrity and thus barrier function, which leads to barrier damage, and consequent inflammation processes in disease conditions (5, 6). Changes of the structure, expression, and distribution of endothelial TJ may cause serious sequels, like edema, diarrhea, septicaemia, and chronic inflammatory diseases (6). The regulations of assembly, disassembly, and maintenance of TJ are partly through the phosphorylation and dephosphorylation of TJ proteins under the action of intracellular phosphatases and kinases, along with several signaling pathways, including protein kinase C (PKC), myosin light chain kinase (MLCK), myosin light chain 2 (MLC-2), and mitogen-activated protein kinases (MAPK) (6–11). With enormous developments in this area, aiming vascular endothelial TJ may play a indispensable role in the treatment of TJ-related pathologies.

*Glaesserella parasuis* (*G. parasuis*) is one of the most important bacteria causing inflammation and damage to pigs (12). The disease caused by this pathogen is Glässer's disease, which is characterized by meningitis, polyserositis, and severe septicaemia (13, 14). It causes significant morbidity and mortality with tremendous commercial losses to the swine industry (15). Because of the poor cross-protection of current vaccines, antimicrobials are commonly used for the control of this disease (16). Nevertheless, the extensive use of antimicrobials is tightly associated with an increase of bacterial resistance (16). Therefore, exploring the pathogenesis of *G. parasuis* and finding effective prevention and controlling measures have become very urgent. Whether *G. parasuis* can cause damage to vascular TJ in piglets has not been investigated in the literature.

Many flavonoids have been demonstrated to exhibit anti-inflammatory property and have protective effects on TJ barrier functions (17–19). Baicalin is a bioactive flavonoid glycoside extracted from the roots of *Scutellaria baicalensis* Georgi. There is sufficient evidence corroborating the notion that baicalin has anti-inflammatory, antibacterial, antioxidant, and antitumor properties, which makes it in clinical use in human and animals (20–23). Previous studies by our group showed that baicalin has anti-inflammatory effects in LPS and *G. parasuis*-challenged piglets by suppressing inflammatory cytokines and HMGB1 through NF-κB and NLRP3 pathway and reversing apoptosis by inhibiting PKC-MAPK pathways (24–27). Besides, baicalin has the capacity to protect TJ in epithelial and endothelial cells (28–30). However, whether baicalin can protect vascular TJ in piglets with *G. parasuis* infection was also unclear. After oral administration, baicalin is poorly absorbed in the intestinal tract for its high polarity and displays enterohepatic recycling and a complex metabolism (31). Our previous research showed that baicalin is highly absorbed and utilized using its sodium salt after intramuscular administration and suitable for treating infectious diseases in piglets (32).

The present work is focused on the vascular TJ damage of *G. parasuis* and the protection effects and its underlying mechanisms of baicalin on vascular TJ, including occludin, ZO-1, claudin-1, and JAM-1. Our results demonstrate that baicalin can be considered a promising natural agent to prevent and control *G. parasuis* infection.

## Materials and Methods

### Ethics Statement

All animal experimental protocols were in accordance with the Wuhan Polytechnic University Laboratory Animals Welfare and Animal Experimental Ethical Inspection (reference number WP20100501).

### Drugs

Baicalin was purchased from National Institutes for Food and Drug Control (B110715-201318, Beijing, China). Sodium baicalin (>95% pure) was synthetized at Hubei key Laboratory of Animal Nutrition and Feed Science (Wuhan, China) (32). Flunixin meglumine (FM) and ethyl pyruvate (EP), used as positive controls, were purchased from Guangdong WenS Dahuanong Biotechnology Co. Ltd. (Yunfu, China) and Shanghai Macklin Biochemical Co. Ltd. (Shanghai, China), respectively.

### Bacterial Strains

*Glaesserella parasuis* (strain SH0165 serovar 5) was isolated from the lung of a infected pig with typical characteristics of Glässer's disease. The strain was cultivated in tryptic soy broth (Difco, USA) at 37°C for 12 h supplemented with fetal bovine serum (Gibco, Australia) and nicotinamide adenine dinucleotide (Sigma, USA).

### Animals

The study was carried out at Animal Experimental Base in Sinopharm Animal Health Corporation Ltd. (Wuhan, Hubei, China). Fifty-six healthy Duroc × Landrace × Large White piglets (weighing 8–10 kg, 23-day weaned) were purchased from Wuhan Wannianqing Animal Husbandry Co. Ltd. (Wuhan, China). They were confirmed negative for *G. parasuis* before the experiment using INgezim Haemophilus antibody test kit (11.HPS.K.1/4, INgezim, Spain).

### Experimental Design

The piglets were divided into seven groups randomly, each group consisting of eight piglets, including control, *G. parasuis*, EP + *G. parasuis*, FM + *G. parasuis*, and baicalin + *G. parasuis* group (25, 50, and 100 mg/kg b.w.). EP was injected intraperitoneally to the piglets in EP + *G. parasuis* group at 40 mg/kg b.w. FM was injected intramuscularly to the piglets in FM + *G. parasuis* group at 2 mg/kg b.w. Sodium baicalin dissolved in saline was administered intramuscularly. Thirty minutes after the drug treatment, all the piglets except those in control group were inoculated intranasally and intraperitoneally with SH0165 (1 × 10^9^ CFU) in 1 ml of normal saline, respectively (33, 34). An equivalent volume of saline was injected intraperitoneally to the piglets in the control group. EP, FM, and baicalin were administered twice daily with an interval of 6 h until the day of postmortem examination. On the 8th day after *G. parasuis* challenge, the piglets were humanely euthanized by intravenous injection of sodium pentobarbital and exsanguination. Aorta samples were carefully collected and stored at −80°C for further experimental processing.

### RNA Extraction and RT-PCR

RNA prep pure Cell/Bacteria Kit (Tiangen, China) was used to isolate the total RNA from aorta homogenates. Reverse Transcription Kit (Takara, Japan) was used to reverse transcribe the RNA into cDNA. Primer 6.0 was used to design the primers for occludin, ZO-1, claudin-1, and JAM-1 (Table 1). The conditions of RT-PCR were as follows: 95°C for 5 min, then followed by 32 cycles of amplification at 95°C for 30 s, Tm temperature for 32 s and 72°C for 30 s, and the final extension was at 72°C for 5 min. Gel Image System (Tanon 4100) was used for the quantification of the densities of each band. The quantitative fluorescence results were counted by 2^−ΔΔCt^ using normalization method.

**Table 1:** Primer sequences for Q-RT-PCR.

**Gene**	**Nucleotide sequences (5′-3′)**		**T_**m**_ (°C)**	**Length (bp)**
occludin	Forward	GAGTGATTCGGATTCTGTCT	50.3	181
	Reverse	TAGCCATAACCATAGCCATAG	50.2	
ZO-1	Forward	GAAGATGATGAAGATGAGGATG	50.3	184
	Reverse	GGAGGATGCTGTTGTCTC	49.9	
claudin-1	Forward	CCTTGCTGAATCTGAACAC	49.5	135
	Reverse	GCACCTCATCATCTTCCAT	50.0	
JAM-1	Forward	TGACAGAACAGGCGAATG	50.1	167
	Reverse	GCAGCATAGGCAGGAATT	50.1	

### Immunofluorescence Microscopy

The distribution patterns of claudin-1, occludin, ZO-1, and JAM-1 proteins in aorta were detected by immunofluorescence. Thin sections of paraffin-embedded aorta were prepared and mounted into adhesive microscopic glass slides. The sections were dewaxed and permeabilized with citrate buffer for 15 min in microwave, washed, and blocked with 5% GSA (diluted in phosphate buffered saline) at room temperature for 1 h. After incubating with rabbit anti-claudin-1, anti-occludin, anti-ZO-1, and anti-JAM-1 antibody (diluted at a 1:100), respectively, at 4°C overnight, the sections were incubated with Cy3-labeled goat anti-rabbit (diluted at 1:100, Beyotime, China) at room temperature for 1 h. A confocal microscope was used to capture fluorescence images (magnification 10 × 40). Image J software version 1.8.0 (National Institutes of Health, Bethesda, MD, USA) was used to semiquantitatively measure fluorescence density in the selected areas to assess the amounts of each TJ protein (35). The image analyses were performed in a blinded manner. The analyst was unaware of how the animal was treated prior to tissue sectioning.

### Western Blot

Protein expressions of PKC-α, PKC-δ, p-PKC-α, p-PKC-δ, MLCK, MLC-2, and p-MLC-2 were detected by Western blotting analysis. Total protein extraction kit (Beyotime, China) and BCA protein assay kit (Sigma, USA) were used to extract the total protein in aorta and measure the protein concentrations. The total proteins were fractionated using 10% SDS-PAGE, transferred onto polyvinylidene fluoride (PVDF) membranes, and then blocked for 3 h with 5% skimmed milk. After incubation with special antibodies (diluted at 1:1,000, including 5% BSA TBS-T, rabbit anti-β-actin, anti-PKC-δ, anti-PKC-α, anti-p-PKC-δ, and anti-p-PKC-α, Cell Signaling, USA; anti-MLCK, anti-MLC-2, and anti-p-MLC-2, Abcam, China) at 4°C for 14 h, corresponding HRP labeled secondary antibodies (diluted at 1:4,000) were added and incubated at 37°C for 3 h. Enhanced chemiluminescent (ECL) reagent (Beyotime, China) was added to determine the protein expression levels, and Azure Bio-imaging systems (California, USA) were used to capture the images. FluorChem FC2 AIC system (Alpha, USA) was used for quantitative analysis. The internal control used was β-actin.

### Statistical Analysis

Data were exhibited as mean ± standard deviation (SD). Statistical analysis was performed using SPSS Statistics 17.0 (IBM, USA). ANOVA was used to assess the differences between the data sets. LSD method was used to perform multiple comparisons between groups. Differences associated with *p* < 0.05 was considered statistically significant.

## Results

### Effects of Baicalin on Expression of Vascular TJ Genes in *G. parasuis* Challenged Piglets

**Figure 1:**
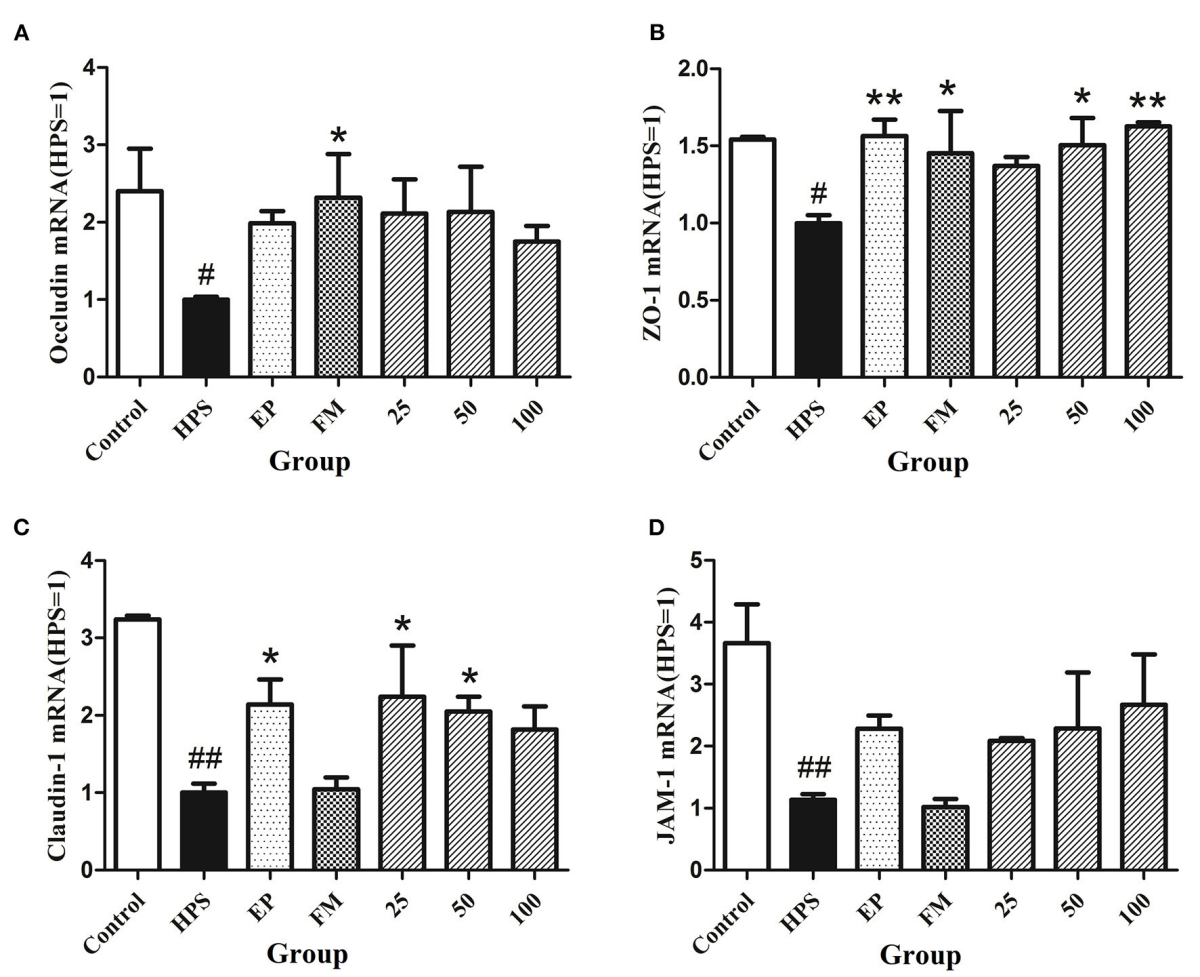
Effects of baicalin on expression of TJ genes occludin **(A)**, ZO-1 **(B)**, claudin-1 **(C)**, and JAM-1 **(D)** in aorta of piglets challenged with *G. parasuis* (mean ± SD, *n* = 3). HPS: *G. parasuis* group, EP: EP + *G. parasuis* group, FM: FM + *G. parasuis* group, 25: 25 mg/kg baicalin + *G. parasuis* group, 50: 50 mg/kg baicalin + *G. parasuis* group, 100: 100 mg/kg baicalin + *G. parasuis* group. ^#^*p* < 0.05 vs. control, ^##^*p* < 0.01 vs. control, *p < 0.05 vs. *G. parasuis* group, **p < 0.01 vs. *G. parasuis* group.

The expressions of vascular TJ genes occludin, ZO-1, claudin-1, and JAM-1 in aorta were detected using RT-PCR. The results showed that *G. parasuis* decreased the mRNA expressions of occludin, ZO-1, claudin-1, and JAM-1, compared with control group (Figure 1). EP could upregulate the expression of each gene. FM could upregulate the gene expression of ZO-1 and occludin. Baicalin could restore the expression of claudin-1, occludin, ZO-1, and JAM-1 to a certain extent in aorta of piglets infected with *G. parasuis*.

### Effects of Baicalin on the Distribution of Vascular TJ in *G. parasuis* Challenged Piglets

**Figure 2:**
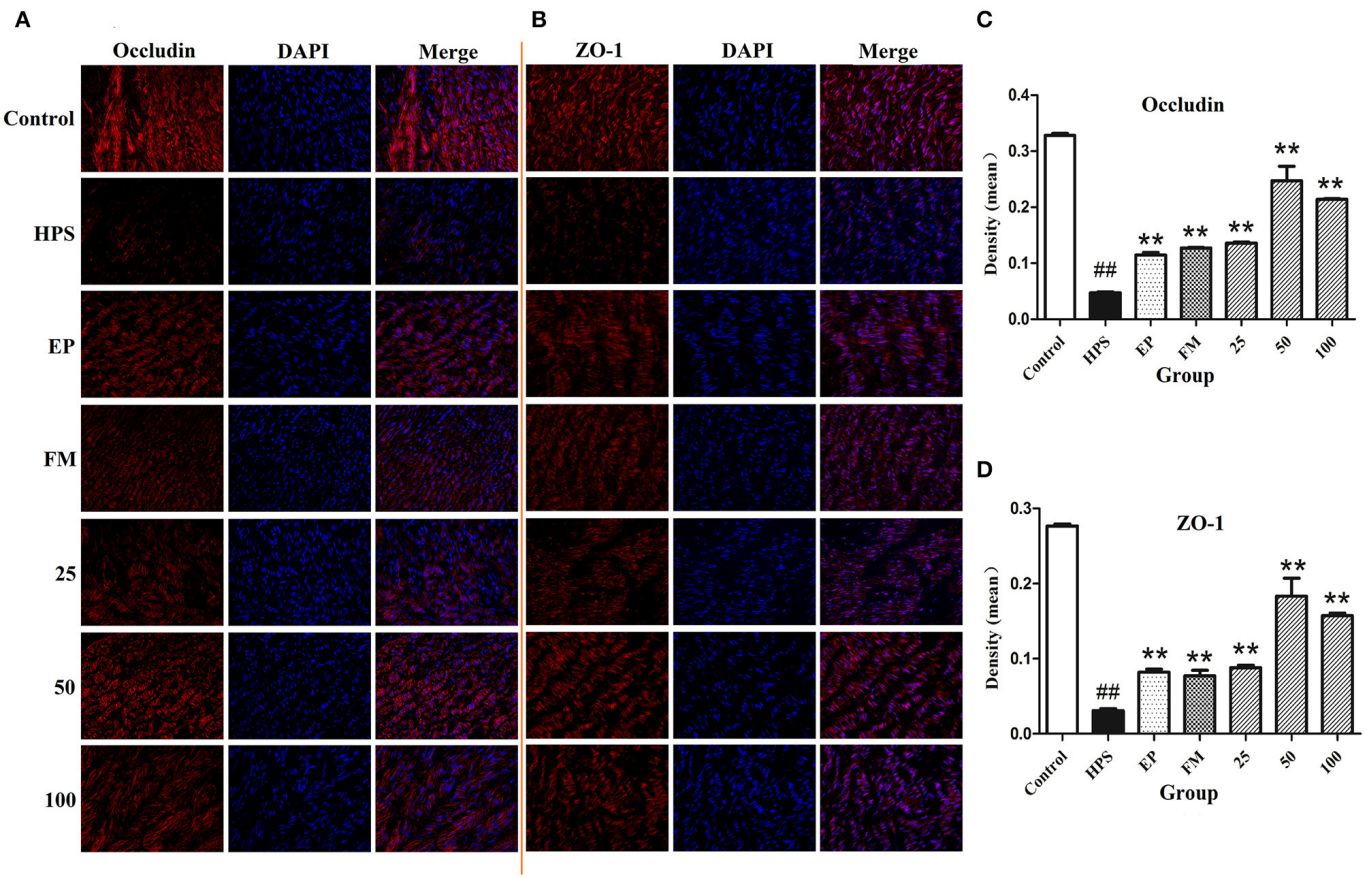
Effects of baicalin on distribution of occludin **(A)** and ZO-1 **(B)** in aorta of piglets challenged with *G. parasuis* (magnification 10 × 40). Fluorescence of occludin **(C)** and ZO-1 **(D)** was measured using Image J. HPS: *G. parasuis* group, EP: EP + *G. parasuis* group, FM: FM + *G. parasuis* group, 25: 25 mg/kg baicalin + *G. parasuis* group, 50: 50 mg/kg baicalin + *G. parasuis* group, 100: 100 mg/kg baicalin + *G. parasuis* group. ^##^*p* < 0.01 vs. control, ** *p* < 0.01 vs. *G. parasuis* group.

**Figure 3:**
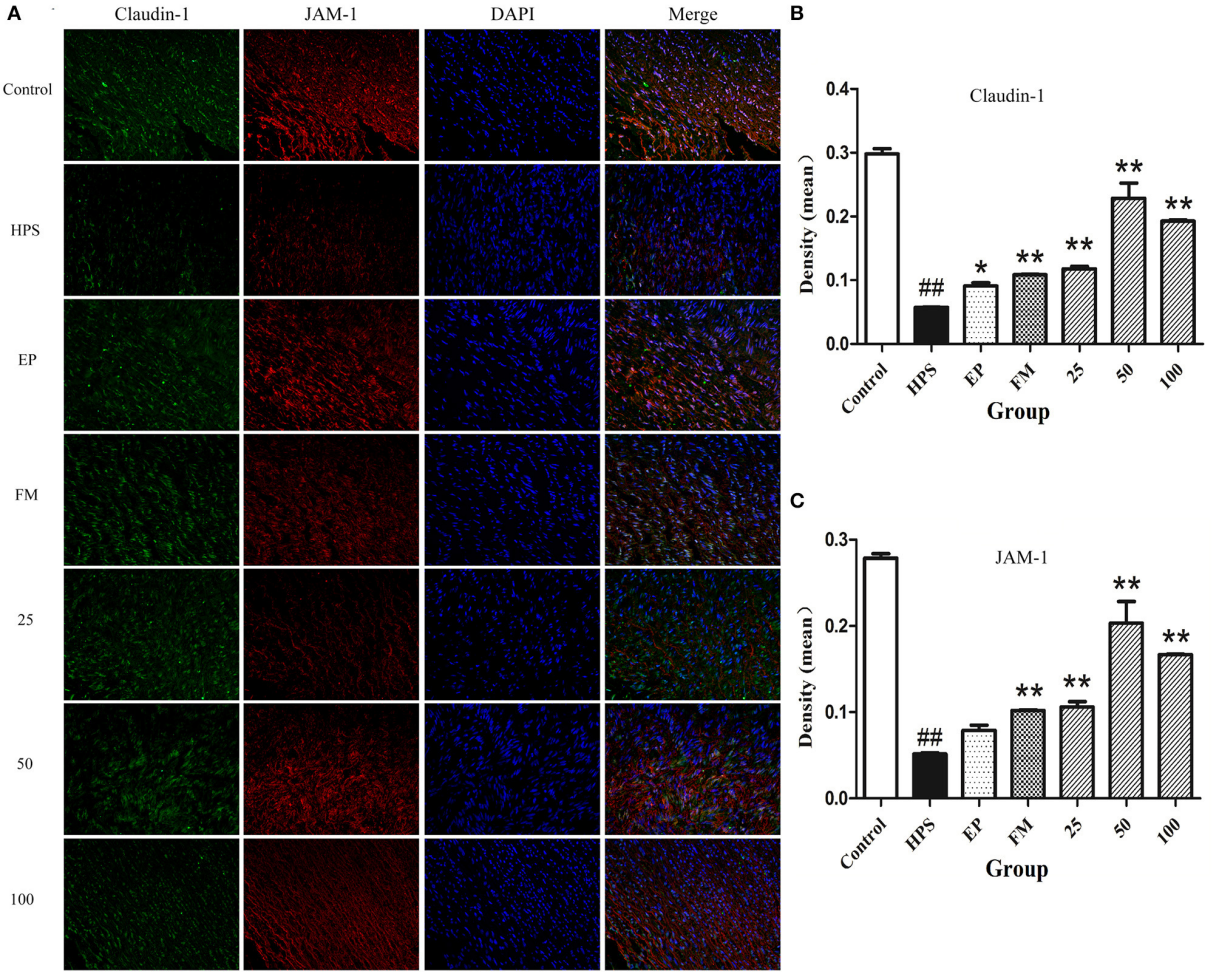
Effects of baicalin on the distribution of claudin-1 and JAM-1 **(A)** in aorta of piglets challenged with *G. parasuis* (magnification 10 × 40). Fluorescence of claudin-1 **(B)** and JAM-1 **(C)** was measured using Image J. HPS: *G. parasuis* group, EP: EP + *G. parasuis* group, FM: FM + *G. parasuis* group, 25: 25 mg/kg baicalin + *G. parasuis* group, 50: 50 mg/kg baicalin + *G. parasuis* group, 100: 100 mg/kg baicalin + *G. parasuis* group. ^##^*p* < 0.01 vs. control, *p < 0.05 vs. *G. parasuis* group, **p < 0.01 vs. *G. parasuis* group.

The organization and distribution images of claudin-1, occludin, ZO-1, and JAM-1 were determined by immunofluorescence (Figures 2A,B, 3A). The fluorescence of TJ in aorta was quantified using Image J by densitometric analysis (Figures 2C,D, 3B,C). *Glaesserella parasuis* infection could significantly alter the distribution patterns of occludin, ZO-1, claudin-1, and JAM-1 in aorta compared to control group. Those proteins staining appeared to be reduced and fragmented by *G. parasuis* infection. EP or FM could attenuate this disorganization. Baicalin (25, 50, and 100 mg/kg) could significantly maintain the continuous organization of TJ and prevent the abnormalities caused by *G. parasuis* (*p* < 0.01).

### Effects of Baicalin on PKC and MLCK/MLC Pathways in *G. parasuis* Challenged Piglets

**Figure 4:**
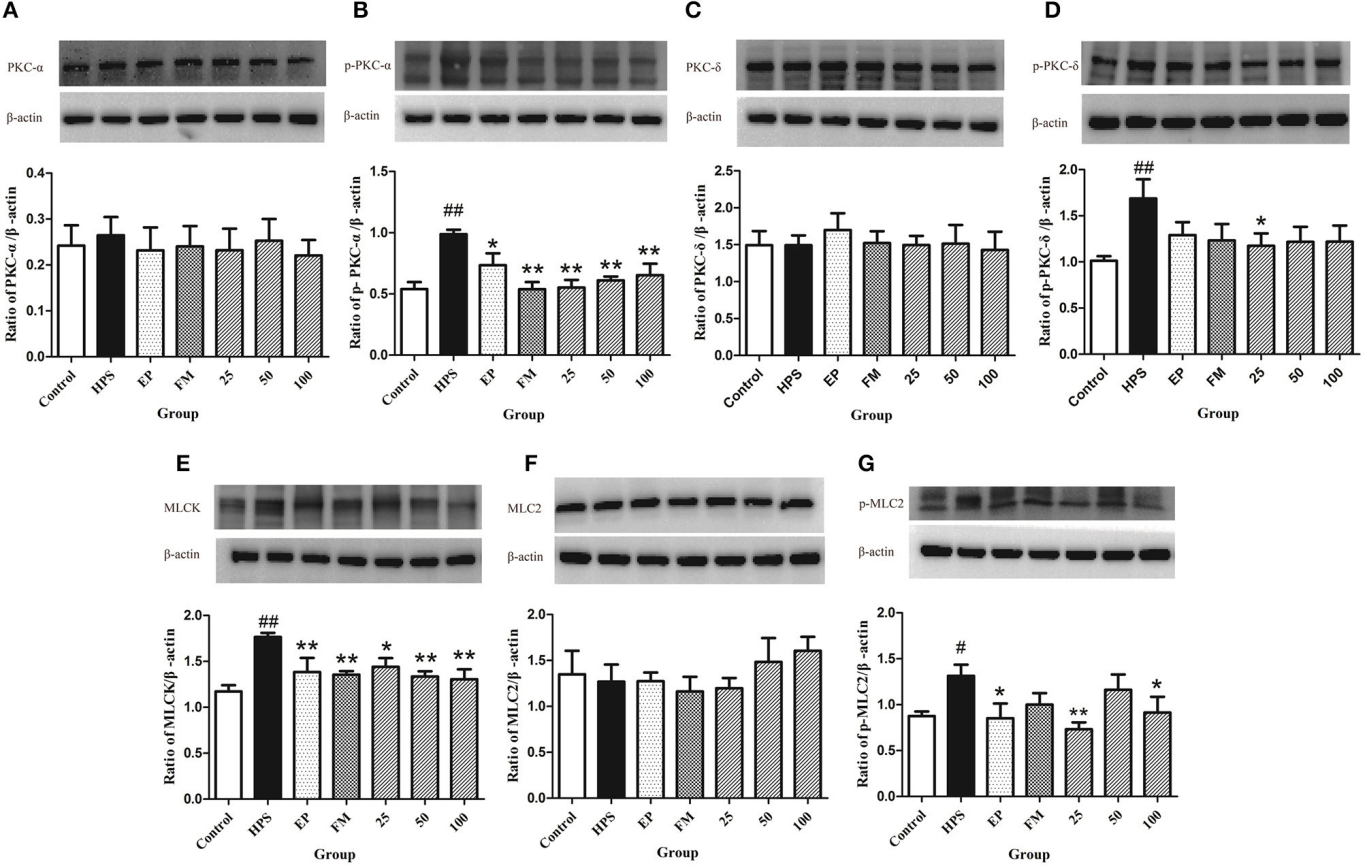
Effects of baicalin on PKC and MLCK/MLC pathways in aorta activated by *G. parasuis* PKC-α **(A)**, p-PKC-α **(B)**, PKC-δ **(C)**, p-PKC-δ **(D)**, MLCK **(E)**, MLC-2 **(F)**, and p-MLC-2 **(G)** (mean ± SD, *n* = 3). HPS: *G. parasuis* group, EP: EP + *G. parasuis* group, FM: FM + *G. parasuis* group, 25: 25 mg/kg baicalin + *G. parasuis* group, 50: 50 mg/kg baicalin + *G. parasuis* group, 100: 100 mg/kg baicalin + *G. parasuis* group. ^#^*p* < 0.05 vs. control, ^##^*p* < 0.01 vs. control, *p < 0.05 vs. *G. parasuis* group, **p < 0.01 vs. *G. parasuis* group.

The protection mechanisms of baicalin on TJ of *G. parasuis* infected piglets were determined by measuring related protein expressions using Western blot assays. The phosphorylation levels of PKC-δ and PKC-α were significantly increased under *G. parasuis* infection (*p* < 0.01), while PKC-δ and PKC-α protein levels remained unchanged (*p* > 0.05). EP, FM, and baicalin could downregulate the expression of p-PKC-α and p-PKC-δ induced by *G. parasuis* and have no effect on PKC-α and PKC-δ protein expression (Figures 4A–D).

The MLCK protein was significantly increased in the aorta in *G. parasuis* group compared to control group (*p* < 0.01). EP and FM could significantly inhibit the expression of MLCK protein. All levels of baicalin significantly altered the effect of *G. parasuis* on the expression of MLCK (*p* < 0.01) (Figure 4E).

The phosphorylation levels of MLC-2 were markedly elevated by *G. parasuis* infection (*p* < 0.01), and the protein levels of MLC-2 remained unified between the control and *G. parasuis* group (*p* > 0.05). EP and 25 and 100 mg/kg baicalin could significantly downregulate the expression of p-MLC-2 and have no effect on the expression of MLC-2 (Figures 4F,G).

## Discussion

*Glaesserella parasuis*, in which pathogenesis relies on its capacity to interplay with invading adjacent tissues and endothelial cells, is responsible for polyserositis and meningitis. Under specific conditions, *G. parasuis* virulent strains can break the mucosal barrier, access the bloodstream, and cause severe septicaemia (36). Once *G. parasui*s has the chance to cross the blood-brain barrier, which was comprised of microvascular endothelial cells, it can get access to the central nervous system and may cause meningitis (37, 38). Evidence showed that *G. parasuis* demonstrated extreme adhesion and invasion properties to porcine aorta endothelial cells and brain microvascular endothelial cells (39), suggesting that *G. parasuis* could damage the vascular barrier. Necropsy results in our previous experiment show that *G. parasuis* caused server pericarditis, peritonitis, meningitis, and septicemia in piglets (27, 40). EP and FM were used as positive controls in the experiment derived from their anti-inflammatory effects to control inflammation-related diseases (27). In the previous and current experiments, FM shows a better effect to alleviate the clinical symptoms of *G. parasuis* infection than EP. Administration of baicalin also demonstrated to attenuate polyserositis and meningitis caused by *G. parasuis*, providing the basis for exploring its mechanism of action on vascular barrier protection.

TJ are multiprotein complexes that constitute a selectively permeable seal between adjoining endothelial or epithelial cells (41). Based on its dynamic structure, TJ are indispensable in conserving the endothelial barrier integrity. Pathogens can directly interact with TJ proteins or indirectly break down the TJ barrier to infect the body by their secreted toxins (42). They can upregulate or downregulate TJ protein expression, redistribute TJ proteins, or break the interactions between proteins to deform TJ structure, break down the barrier, lose cell polarity, and lead to more serious diseases (43). The expression of occludin involves the co-expression of junctional protein ZO-1 in endothelial cell surface (44). Occludin is a key regulator of the TJ barrier, and multiple domains of occludin are involved in regulating cell bypass permeability (45). Lack of occludin can cause moderate TJ dysfunction or of other cellular signaling pathway dysfunction (46). ZOs are intracellular ingredients of TJ, which relate to cortical actin. ZO proteins interact directly with most transmembrane proteins located at TJ. Inflammatory injury can cause abnormal distribution, reduce the expression and dissolution of ZO-1 protein, and damage the TJ structure between cells (47). Transmembrane protein JAMs can interact with adjacent endothelial cells to stabilize the endothelial barrier. In case of inflammation and ischemia, JAM expression is upregulated and redistributed away from TJ (48).

However, there are little data available on how TJ changes under *G. parasuis* infection and their interaction mechanism. To our knowledge, this is the first study where vascular TJ protein alterations in *G. parasuis* infected piglets were explored. *Glaesserella parasuis* infection caused significant downregulation of vascular TJ mRNA expression. The distributions of occludin, ZO-1, claudin-1, and JAM-1 protein in aorta of *G. parasuis* infected piglets were significantly disrupted. These data provide the evidence that *G. parasuis* infection can alter the vascular TJ and damage the vascular barrier. Baicalin can alleviate the downregulation of each TJ mRNA in aorta. Baicalin can prevent the abnormalities caused by *G. parasuis* and maintain the continuous organization of vascular TJ. These findings identify a *G. parasuis*-induced impairment of vascular TJ integrity, a process largely prevented by baicalin supplementation.

PKC isoforms are important regulators of junctional permeability. Studies have shown that PKCs can regulate endothelial and epithelial barriers *via* the direct phosphorylation of TJ proteins and their regulatory effects as intracellular signaling molecules (6). Endothelial permeability can be increased by PKC activator phorbol ester and decreased by PKC inhibitor (2). PKC mediates the phosphorylation of occludin and its regulation in cell distribution. PKC is involved in the translocation of ZO-1 from the cell interior to cell membranes, which can affect the regulation and formation of TJ. PKC-α has a crucial role in mediating endothelial TJ disassembly (49). PKC-δ is a “novel” PKC isoform. Overexpression of PKC-δ decreases endothelial permeability by increasing focal adhesion contacts (50).

The changes of PKC-α and PKC-δ in the aorta of piglets infected with *G. parasuis* were detected in the current study. The expression of phosphorylation of PKC-α and PKC-δ in aorta of *G. parasuis* infected piglets was significantly increased, while no obvious change was found in non-phosphorylated PKC-α and PKC-δ compared with the control group, indicating that PKC-α and PKC-δ in aorta was activated by *G. parasuis*. Baicalin can alleviate the phosphorylation of PKC-α and PKC-δ, which is in consistent with our previous work and the results of other studies of the regulation effects of baicalin on PKC (26, 40, 51–53). The results suggest that the protective effect of baicalin on vascular TJ may be related to inhibit PKC or the downstream pathways.

MLCK and its regulation of MLC have been demonstrated to be the most important factors that influence TJ during inflammation (54). MLCK is substantial for the reorganization of the cytoskeleton, which result in disruption of barrier integrity (55). Activated MLCK leads to the reorganization of TJ proteins, which further triggers the endocytosis of TJ protein and changes of barrier (42). Increased MLCK is an indicator of TJ barrier disruption induced by pro-inflammatory cytokines. TJ proteins can be regulated by MLC-2, which principally depends upon the activation of MLCK (10, 11, 56).

Our results showed that the MLCK and p-MLC-2 protein were significantly increased in the peritoneum in *G. parasuis* challenged piglets, suggesting that *G. parasuis* infection could cause TJ barrier disruption. Baicalin obviously inhibited the protein levels of MLCK and p-MLC-2 induced by *G. parasuis*. The results indicate that the protective effects of baicalin on TJ may derive from inhibiting MLCK/MLC pathway. In order to comprehend the interaction mechanisms between *G. parasuis* and vascular TJ and the protective effect of baicalin on it, further studies need to be carried out.

In summary, our data provide ample evidence that *G. parasuis* can affect vascular endothelial barrier by downregulating vascular TJ expression and disrupting the distribution of TJ proteins. Baicalin can protect vascular TJ from *G. parasuis*-induced injury. It is possible that the protective effects of baicalin on vascular TJ are closely related to the inhibition of the activation of PKC and MLCK/MLC pathways. It is of great significance that baicalin should be listed as a promising drugs for the treatment of Glässer's disease.

## Data Availability Statement

The original contributions presented in the study are included in the article/supplementary material, further inquiries can be directed to the corresponding author/s.

## Ethics Statement

The animal study was reviewed and approved by Wuhan Polytechnic University Laboratory Animals Welfare and Animal Experimental Ethical Inspection.

## Author Contributions

YL and YQ: study conception. YL, XL, and ZZ: study design, writing, and editing. XL and ZZ: experiment coordination. JX and JZ: sample collection. JZ and CY: laboratory work performing. SF and ZW: data analysis. YL, C-AH, and YQ: project direction. All authors contributed to the article and approved the submitted version.

## Conflict of Interest

The authors declare that the research was conducted in the absence of any commercial or financial relationships that could be construed as a potential conflict of interest.
